# Bilateral Iris Mammillations in Amblyopic Eyes without Oculodermal Melanocytosis or Neurofibromatosis

**DOI:** 10.1155/2018/2534042

**Published:** 2018-10-29

**Authors:** Megumi Yamamoto, Tatsuya Mimura, Koichi Matsumoto, Shigeki Hamano, Hisataka Nanba, Shoko Ubukata, Emiko Watanabe, Atsushi Mizota

**Affiliations:** Department of Ophthalmology, Teikyo University School of Medicine, Tokyo 173-8605, Japan

## Abstract

**Purpose:**

Iris mammillations are related to oculodermal melanosis and iris nevi. We report a rare case of bilateral simple iris mammillations without ocular melanosis or systemic neuronal disorders.

**Case Report:**

A healthy 10-year-old Japanese girl was found incidentally to have bilateral iris mammillations while being treated for amblyopia. The best-corrected visual acuity was 20/40 in both eyes. Ocular examination showed evenly spaced, uniform-size, iris protrusions completely covering the iris surface bilaterally. There were no other ocular or neurological abnormalities.

**Conclusion:**

To the best of our knowledge, this is the first report of bilateral iris mammillations in Japan. Our case emphasizes that iris mammillations can occur even without ocular melanocytosis or systemic diseases.

## 1. Introduction

Iris mammillation is an extremely rare ocular disorder in which small papilliform nodules are seen in part or the entire iris surface of one eye or both eyes [1]. These nodules usually are located on the surface of a brown iris or on iris nevi. Iris mammillations are shaped like Lish nodules that are seen in patients with neurofibromatosis (NF). Thus, most of the iris nodules can be accompanied by NF type I, phakomatosis pigmentovascularis (PPV) type IIb, and oculodermal melanocytosis [1–7]. Iris mammillations are seen more often in highly pigmented ethnic groups than in Caucasians or Asians [1, 8–11], and it has never been reported in Japan. We report an extremely rare case of bilateral iris mammillations without other ocular or neural abnormalities in a young Japanese patient.

## 2. Case Presentation

A 10-year-old Japanese girl was referred to our hospital with a six-year history of bilateral amblyopia. The patient had undergone complete ophthalmological examinations and evaluations by many ophthalmologists at several medical institutions because of her visual disorder since she was a preschooler. However, the cause of visual disturbance was not determined, and the patient was tentatively diagnosed with amblyopia or visual disturbances of psychogenic origin. She was examined regularly at 2 to 3 months' intervals since the first evaluation. The patient had never been diagnosed with iris-related diseases such as iris nodules.

**Figure 1:**
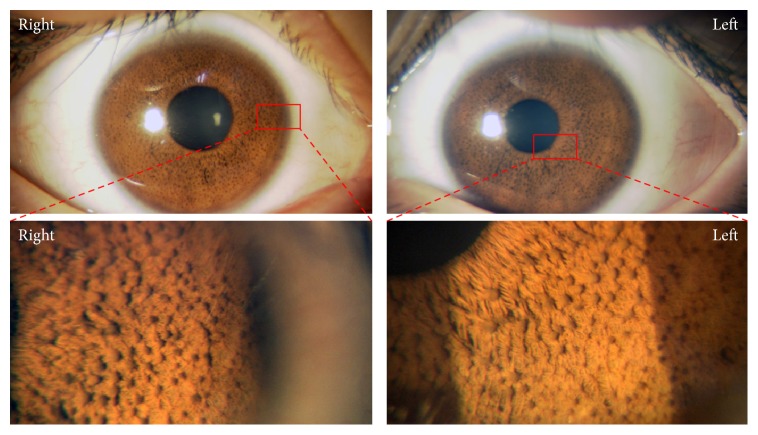
Slit-lamp photomicrographs of the anterior segment of both eyes at the first visit.

**Figure 2:**
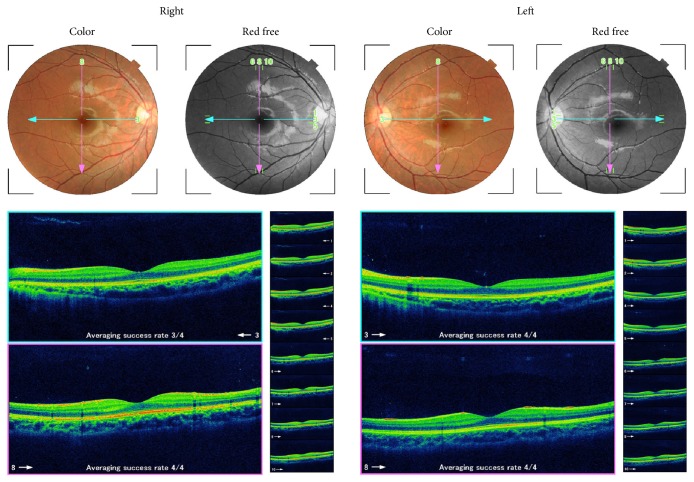
Fundus photographs and optical coherence tomographic image of both eyes.

At the first examination at our hospital, her best-corrected visual acuity (BCVA) was 20/40 in the right eye and 20/40 in the left eye. The pupils were of equal size and there was no afferent pupillary defect. Slit-lamp examination revealed numerous small iris nodules bilaterally (Figure 1). Extraocular movements were full without nystagmus. The intraocular pressure was 12 mmHg in the right eye and 11 mmHg in the left eye. The ophthalmoscopic findings of the retina were within the normal limits, and optical coherence tomography showed that the macula appeared normal in both eyes (Figure 2). Her family had no similar iris anomaly. The visual field determined by Humphrey program 30-2 and color vision test were within normal limits in both eyes. The patient was prescribed spectacle correction of +0.25 -0.25 x130 in the right eye and +0.25 -0.25 x160 in the left eye to reduce the risk of amblyopia. After 3 years of treatment, the BCVA had improved to 20/25 in the right eye and 20/20 in the left eye. During the treatment of amblyopia, neuronal complications such as mental disease, neuropathy, and neurofibromatosis were not observed.

## 3. Discussion

Iris mammillations are extremely rare and have never been reported in a Japanese individual. A summary of the case reports regarding iris mammillations is presented in Table 1. The differential diagnosis of iris mammillations is from Lisch nodules, iris nevi, iris melanoma, Brushfield flecks, retinoblastoma, and the Cogan-Reese (ICE) syndrome [1–7]. In our patient, the shapes of the iris nodules were important for the diagnosis of iris mammillations. This case indicates the importance of suspecting a diagnosis of iris mammillations in any patient presenting with a large number of uniform and diffuse nodules that cover the entire surface of the iris.

Ragge et al. suggested that iris mammillations have been confused with Lisch nodules associated with NF1 which was previously known as von Recklinghausen disease [1, 2]. Lisch nodules, also known as iris hamartoma, are irregularly spaced, pigmented brown hamartomatous nodular aggregates of dendritic melanocytes [1, 6]. They are accompanied by ocular hypertension or intraocular malignancy, and they are also associated with external ocular manifestations such as oculodermal melanosis [1, 6]. The iris nodules in our case were protruding nodules and had the same color as those seen in dark brown irides. Furthermore, our patient had no sign of ocular melanocytosis. Thus, iris mammillations in our case could be easily distinguished from Lisch nodules which are well-defined dome-shaped nodules by slit-lamp examination [1].

In conclusion, this is the first report of bilateral iris mammillations detected by chance in a child who was being treated for amblyopia in Japan. There may be still many people with this disorder without an accurate diagnosis because these patients are generally asymptomatic and may not visit hospital. Ophthalmologists should be aware that iris mammillations can occur independently of other manifestations such as dark pigmentation changes within the iris, melanosis, or systemic diseases.

## Figures and Tables

**Table 1:** Modified table from Ragge et al. (1996) summarizing the case reports of iris mammillations [1].

**No**	**Age/sex**	**Ethnic origin**	**Ocular involvement**	**Iris involvement**	**Melanosis oculi**	**Naevus**	**Ocular disease**	**Other disease**	**Journal**	**Year**	**Author**
**1**	8F	Caucasian	U	P	N	Y	Myopia	None	Eye	1996	Ragge

**2**	8F	Hispanic	B	T	N	N	High myopia	CHD, cleft palate, marfanoid habitus	Eye	1996	Ragge

**3**	9M	Hispanic	U	P	Y	Y	Esotropia and amblyopia of other eye	Seizures	Eye	1996	Ragge

**4**	20M	Asian-Indian	B	P	Y	N	Iris hamartomas	Neurofibromatosis type 1	Eye	1996	Ragge

**5**	7F	Hispanic	B	ST	N	N	Optic neuritis	TB frontal arachnoid cyst	Eye	1996	Ragge

**6**	10F	Middle Eastern	B	T	Y	N	Calcified ciliary body mass	Mother also had bilateral iris elevations	Eye	1996	Ragge

**7**	5F	Hispanic	B	T	Y	N	Congenital glaucoma	Phakomatosis pigmentovascularis Iib	Eye	1996	Ragge

**8**	28M	Asian (Indian)	B	ST	N	N	None	None	Eye	1996	Ragge

**9**	2M	N. African	B	T	Y	N	None	Ectopic Mongolian spot, preauricular skin tag, abnormal rib	Eye	1996	Ragge

**10**	8F	-	U	T	Y	N	Choroidal melanoma, Retinal detachment	None	Arch Ophthalmol	2000	Gündüz

**11**	1M	Caucasian	B	-	N	N	None	None	J Fr Ophtalmol	2006	Kharrat

**12**	8M	Caucasian	B	-	N	N	None	None	J Fr Ophtalmol	2006	Kharrat

**13**	5M	Caucasian	U	-	Y	N	Ipsilateral ocular melanocytosis	None	J Fr Ophtalmol	2006	Kharrat

**14**	9F	Türkler	B	P	N	N	Optic nerve damage, Persistent pupillary membrane	None	Clin Exp Optom	2007	Ozdamar

**15**	7F	Asian (Malaysian)	B	T	N	N	None	Siblings, CAH, Clitoris acne, Labioscrotal hyperpigmentation	BMJ Case Reports	2010	Peyman

**16**	7F	Asian (Malaysian)	B	T	N	N	None	Siblings, CAH, Clitoris acne, Labioscrotal hyperpigmentation	BMJ Case Reports	2010	Peyman

**17**	5M	Caucasian	U	T	Y	N	Scleral pigmentation	None	Arch Soc Esp Oftalmol	2014	Marugán B

**18**	8F	Hispanic	B	T	N	N	None	None	Arch Soc Esp Oftalmol	2014	Marugán B

**19**	16F	Caucasian	B	T	N	N	None	None	Arch Soc Esp Oftalmol	2014	Marugán B

**20**	22F	Hispanic	B	P	Y	N	Eyelid trichilemmoma	Twins, Cowden syndrome	JAAD Case Reports	2016	Suaiti

**21**	22F	Hispanic	B	P	Y	N	Eyelid trichilemmoma	Twins, Cowden syndrome	JAAD Case Reports	2016	Suaiti

**22**	12M	Hispanic	B	ST	Y	Y	Monolateral ocular melanocytosis, Glaucoma, Choroidal haemangioma	Sturge-Weber Syndrome, naevus flammeus of the face	Csse Rep Ophthalmol	2017	Plateroti

**23**	10F	Asian, (Japanese)	B	T	N	N	Amblyopia	None	Case Rep Ophthalmol Med	This Case	Yamamoto, Mimura

Age, years; M, male; F, female; U, unilateral; B, bilateral; P, partial; T, total; ST, subtotal; Y, yes; N, no; CHD, congenital heart disease; CAH, congenital adrenal hyperplasia.
